# Genetic Engineering of *Lactococcus lactis* Co-producing Antigen and the Mucosal Adjuvant 3′ 5′- cyclic di Adenosine Monophosphate (c-di-AMP) as a Design Strategy to Develop a Mucosal Vaccine Prototype

**DOI:** 10.3389/fmicb.2018.02100

**Published:** 2018-09-04

**Authors:** Ingrid Quintana, Martín Espariz, Silvina R. Villar, Florencia B. González, Maria F. Pacini, Gabriel Cabrera, Iván Bontempi, Estefanía Prochetto, Jörg Stülke, Ana R. Perez, Iván Marcipar, Victor Blancato, Christian Magni

**Affiliations:** ^1^Laboratorio de Fisiología y Genética de Bacterias Lácticas, Instituto de Biología Molecular y Celular de Rosario (IBR, CONICET UNR), Universidad Nacional de Rosario, Rosario, Argentina; ^2^Department of General Microbiology, GZMB, Georg-August-Universität Göttingen, Göttingen, Germany; ^3^Laboratorio de Biotecnología e Inocuidad de los Alimentos, Facultad de Ciencias Bioquímicas y Farmacéuticas – Municipalidad de Granadero Baigorria (UNR), Rosario, Argentina; ^4^Instituto de Inmunología Clínica y Experimental de Rosario (IDICER, CONICET UNR), Rosario, Argentina; ^5^Facultad de Ciencias Médicas, Centro de Investigación y Producción de Reactivos Biológicos, Universidad Nacional de Rosario, Rosario, Argentina; ^6^Laboratorio de Tecnología Inmunológica, Universidad Nacional del Litoral, Santa Fe, Argentina; ^7^Facultad de Ciencias Médicas, Universidad Nacional del Litoral, Santa Fe, Argentina

**Keywords:** *Lactococcus lactis*, c-di-AMP adjuvant, live vaccine, delivery system, *T. cruzi*, trans-sialidase

## Abstract

*Lactococcus lactis* is a promising candidate for the development of mucosal vaccines. More than 20 years of experimental research supports this immunization approach. In addition, 3′ 5′- cyclic di-adenosine monophosphate (c-di-AMP) is a bacterial second messenger that plays a key role in the regulation of diverse physiological functions (potassium and cellular wall homeostasis, among others). Moreover, recent studies showed that c-di-AMP has a strong mucosal adjuvant activity that promotes both humoral and cellular immune responses. In this study, we report the development of a novel mucosal vaccine prototype based on a genetically engineered *L. lactis* strain. First, we demonstrate that homologous expression of *cdaA* gen in *L. lactis* is able to increase c-di-AMP levels. Thus, we hypothesized that *in vivo* synthesis of the adjuvant can be combined with production of an antigen of interest in a separate form or jointly in the same strain. Therefore, a specifically designed fragment of the trans-sialidase (TScf) enzyme from the *Trypanosoma cruzi* parasite, the etiological agent of Chagas disease, was selected to evaluate as proof of concept the immune response triggered by our vaccine prototypes. Consequently, we found that oral administration of a *L. lactis* strain expressing antigenic TScf combined with another *L. lactis* strain producing the adjuvant c-di-AMP could elicit a TS-specific immune response. Also, an additional *L. lactis* strain containing a single plasmid with both *cdaA* and *tscf* genes under the Pcit and Pnis promoters, respectively, was also able to elicit a specific immune response. Thus, the current report is the first one to describe an engineered *L. lactis* strain that simultaneously synthesizes the adjuvant c-di-AMP as well as a heterologous antigen in order to develop a simple and economical system for the formulation of vaccine prototypes using a food grade lactic acid bacterium.

## Introduction

*Lactococcus* *lactis* is one of the most frequently used microorganisms in the food industry across the world (de Vos, 2011; Smid and Kleerebezem, 2014). Moreover, recent reports that use *L. lactis* as a therapeutic agent for the treatment of different human and animal diseases have stimulated the interest of this microorganism by the pharmaceutical industry. The potential biotechnological applications of this microorganism in the pharmaceutical drug production and the spectrum of possibilities it offers constitutes nowadays one of the most striking reasons for the investigation on *L. lactis* genetic manipulation (Cano-Garrido et al., 2015). In particular, the use of *L. lactis* as a live non-invasive mucosal vaccine seems a promising alternative due to their GRAS (Generally Recognized As Safe) status (Miyoshi et al., 2002; Bermudez-Humaran et al., 2003; Foligne et al., 2007; Wells and Mercenier, 2008; Cano-Garrido et al., 2015; Kim et al., 2015; Mancha-Agresti et al., 2017).

*L. lactis* has been successfully employed to produce specific viral and bacterial antigens to cope infections or non-antigenic immunomodulatory proteins like cytokines or proteases to control infections or more complex inflammatory diseases such as the inflammatory bowel disease (Miyoshi et al., 2002; Bermudez-Humaran et al., 2003; Foligne et al., 2007; Wells and Mercenier, 2008; Marelli et al., 2011; Cano-Garrido et al., 2015; Kim et al., 2015; Mancha-Agresti et al., 2017). Most importantly, it has been used for the expression and delivery of heterologous antigens to develop oral and mucosal vaccines (Wells and Mercenier, 2008; Cano-Garrido et al., 2015).

On the other hand, a key factor for the development of human subunit vaccines is to define not only a suitable antigen but also an adequate adjuvant. Vaccine adjuvant categories are classically based on the underlying mechanism of action. In this regard, they may be divided into delivery systems (or particulate adjuvants) and immune potentiators (or immune stimulators) (Pashine et al., 2005; Wells and Mercenier, 2008). Moreover, both types of adjuvants can act as mucosal adjuvants (Apostolico et al., 2016).

In the last decade, cyclic-di-nucleotides have emerged as promising vaccine adjuvants (Ebensen et al., 2007; Chen et al., 2010; Libanova et al., 2010). In particular, it was demonstrated that 3′ 5′-cyclic-di-adenosine monophosphate (c-di-AMP) promotes the immune response in both human immune cells as well as in mice models (Ebensen et al., 2017). This metabolite is a bacterial second messenger involved in different metabolic processes including potassium uptake, cell turgor and cell wall homeostasis (Reuss et al., 2017; Commichau et al., 2018). Furthermore, recent studies showed that c-di-AMP has a strong mucosal adjuvant activity that potentiates both humoral and cellular immune responses (Chen et al., 2010; Ebensen et al., 2011). In mammals, c-di-AMP is an agonist of the STimulator of INterferon Genes (STING) response, which acts as an innate immune sensor of microbes leading to type I interferon production (Lirussi et al., 2017). Moreover, several investigations have shown that mucosal immunization with c-di-AMP promotes a strong Th1 bias, a requisite for the control of intracellular pathogens (Burdette et al., 2011). Recently it was described that type I IFN is essential for c-di-AMP mediated cross-presentation by a cathepsin independent and TAP and proteasome dependent cytosolic antigen processing pathway, indicating that type I IFN signaling is critical for cyclic di nucleotides-mediated cross-presentation (Lirussi et al., 2017).

In this report we take advantage of the potential of c-di-AMP as mucosal immunostimulator to develop a mucosal vaccine prototype based on an engineered *L. lactis* carrying genes encoding the cyclase enzyme responsible for c-di-AMP synthesis (CdaA) (Reuss et al., 2017), as well as, a specifically designed peptide derived from the trans-sialidase enzyme (TS), a proved immunogenic antigen of the *Trypanosoma cruzi* parasite, the etiological agent of Chagas disease (Nardy et al., 2016). *T. cruzi* TS catalyzes the transfer of sialic acid from the host glycoconjugates to the terminal β-galactopyranosyl residues of mucin-like molecules on the cell surface of parasite (Freire-de-Lima et al., 2015). TS is also involved in different pathways leading to parasite infection and down-regulation of the host immune response. In addition, this antigen is considered nowadays one of the best candidates for the development of prophylactic vaccines against *T. cruzi* (Bontempi et al., 2017). In this study, we constructed a novel engineered *L. lactis* strain able to produce simultaneously the TScf antigen and the c-di-AMP adjuvant. Three successive oral immunizations with this engineered *L. lactis* elicited a clear response against TScf. These results suggest that oral formulations based on both c-di-AMP and heterologous antigen-producing *L. lactis* strain could be used as a new vaccine delivery system aiming to develop specific immune protection.

## Materials and Methods

### Bacterial Strains and Growth Conditions

*Lactococcus* strains were routinely grown in M17 medium (Oxoid) supplemented with 0.5% (wt/vol) glucose (M17G) at 30°C without shaking. Initial pH was adjusted to 7.0 or 5.5 with HCl when specified. *Escherichia coli* strains were used as cloning host. Cultures of *E. coli* were grown aerobically in Luria Bertani medium (LB) at 37°C, and transformed as previously described (Sambrook and Russell, 2001). Agar (1.5%) was added to the medium when was required. Antibiotics were added as selective agents when needed: 5 μg/ml erythromycin and 10 μg/ml chloramphenicol for *L. lactis* and 100 μg/ml ampicillin or 150 μg/ml erythromycin for *E. coli*. Plasmids and bacterial strains used in this study are listed in **Table 1**.

**Table 1:** Plasmids and bacterial strains used in this study.

Strain or plasmid	Description	Reference
**Plasmids**		
pNZ8048	Expression vector containing Pnis nisin -inducible promoter, Cm^R^	de Ruyter et al., 1996
pBV153	Expression vector derived from pBM01 containing chromosomal pH-controllable promoter region Pcit and *Nde*I cloning site, Cm^R^	Marelli and Magni, 2010
pBVGh	Thermosensitive vector derivate of pWV01 for quick generation of gene deletion, Em^R^	Blancato and Magni, 2010
pUC57-TScf	pUC57 derived plasmid encoding codon optimized 6xhis tagged trans-sialidase fragment (TScf).	This work
pNZ-TScf	pNZ8048 derivative carrying the *tscf* gene under Pnis promoter.	This work
pIQ095	pBVGh derivative plasmid carrying the upstream and downstream DNA fragments of *L. lactis gdpP* for gene deletion, Em^R^	This work
pIQ101	pBV153 derivative plasmid carrying *L. lactis cdaA* gene under Pcit promoter, Cm^R^	This work
pIQ10-TS	pBV153 derived plasmid carrying *cdaA* gene under Pcit promoter and trans-sialidase fragment under Pnis promoter, Cm^R^	This work
**Strains**		
*E. coli* DH5-α.	F- ϕ80d/lacZΔM15 Δ(lacZYA-argF) U169 recA1 endA1 hsdR17 (rK-, mK+) phoA supE44 λ- thi- 1 gyrA96 relA1	Hanahan, 1983
*E. coli* EC101	Kan^R^ supE thi Δ(lacproAB) (F′ traD36 proAB lacIq ZΔM15) repA.	Law et al., 1995
*L. lactis* IL1403	Trp+ plasmid-free	Bolotin et al., 2001
*L. lactis* NZ9000	*L. lactis* MG1363 containing *nisRK* genes integrated into pepN locus, plasmid free	Kuipers et al., 1998
*L. lactis* NZ9000 clpP-htrA	NZ9000 carrying *clpP* and *htrA* disruption, plasmid free	Cortes-Perez et al., 2006
*L. lactis* LL0	*L. lactis* IL1403 carrying pBV153 vector	This work
*L. lactis* LL1	*L. lactis* IL1403 carrying plasmid pIQ101	This work
*L. lactis* LL2	*L. lactis* IL1403 ΔgdpP1	This work
*L. lactis* LL3	NZ9000 clpP-htrA carrying pNZ8048 vector	Cortes-Perez et al., 2006
*L. lactis* LL4	*L. lactis* NZ9000 strain harboring pNZ-TScf plasmid, for antigen expression	This work
*L. lactis* LL5	NZ9000 clpP-htrA carrying plasmid pNZ-TScf	This work
*L. lactis* LL6	NZ9000 clpP-htrA carrying plasmid pIQ101	This work
*L. lactis* LL7	NZ9000 clpP-htrA carrying plasmid pIQ10-TS	This work

*Cm^R^ and Em^R^ indicate resistant to chloramphenicol and erythromycin, respectively.*

### Trans-Sialidase Antigen Prediction and TS Fragment Encoding Gene Synthesis

Full sequence of the trans-sialidase enzyme was analyzed in order to predict T epitopes against H-2Kd MHC-I by using the Propred I prediction server (Singh and Raghava, 2001). The gene that encodes the selected fragment of TS (TScf) was synthetized taking into account the codon usage of *L. lactis* MG1363 strain and cloned in pUC57 by GenScript (United States).

### DNA Manipulation and Construction of Recombinant *L. lactis* Strains

**FIGURE 1:**
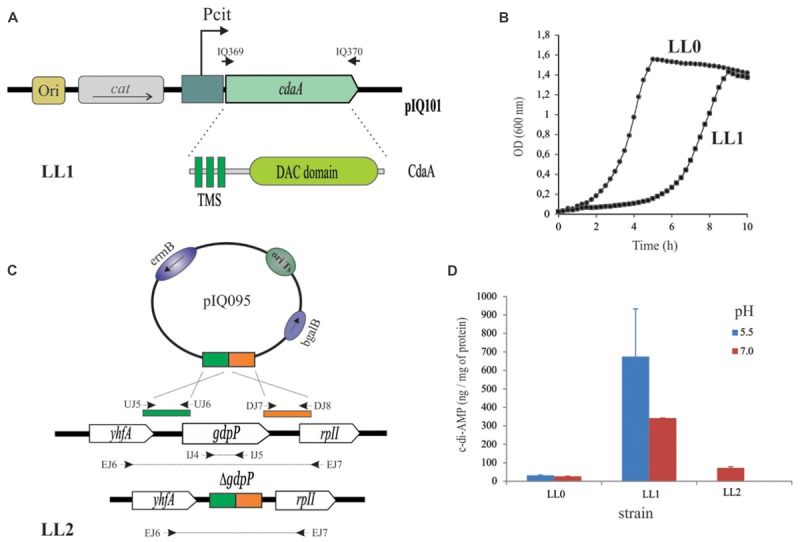
Engineering of *L. lactis* strains with increased cytosolic c-di-AMP levels. **(A)** Schematic map of the recombinant plasmid pIQ101 carrying the membrane lactococcal *cdaA* gene under the pH-controlled Pcit promoter. IQ369 and IQ370 oligonucleotides used for amplification: are indicated in the text. CdaA of *L. lactis* is composed of three transmembrane segments (TMS) and one cytosolic cyclase domain (DAC domain). *cat*: gene encoding chloramphenicol acetyl transferase conferring Cm^R^ phenotype. **(B)** Growth patterns of *L. lactis* IL1403 strain transformed with pBV153 vector (LL0 strain, indicated in circles) or pIQ101 (LL1 strain, indicated by squares). Lactococcal cells were grown in M17G and monitored by OD_600_ measurements for 10 h at 30°C. **(C)** Construction of the deficient *gdpP* phosphodiesterase *L. lactis* strain. Primers indicated by arrows were used for deletion check, see details in the text and (Blancato and Magni, 2010). **(D)** c-di-AMP intracellular levels of *L. lactis* strains. IL1403 derived strain transformed with pNZ8048 (LL0) or pIQ101 (LL1) and LL2 with a deletion in *gdpP* gene. pH 5.5 are indicated in red bar and pH 7.0 in blue.

*cdaA* was amplified by PCR using chromosomal DNA extracted from *L. lactis* IL1403 as template and the pair of primers IQ369 (ACGTAACCATATGTTGACCGACTTCAATC, underlined nucleotides indicate the *Nde*I site) and IQ370 (GCTCTAGAAAGCTTTTATTTGCCATTTTTC, underlined nucleotides indicate the *Xba*I site). The resulting fragment was purified, digested with *Nde*I and *Xba*I and ligated into the *Nde*I-*Spe*I sites of pBV153 vector, originating pIQ101 plasmid (**Figure 1A**). This construction was transformed in *E. coli* DH5α and the primary sequence of *cdaA* gene was checked by sequencing (University of Maine, DNA sequencing Facility, United States). Plasmid pIQ101 was then electroporated in *L. lactis* cells as previously was described (Dornan and Collins, 1990) resulting in strain LL1 (**Table 1**).

*L. lactis gdpP*-defective strain was constructed by gene deletion using the thermosensitive suicide plasmid pIQ095 (derived from vector pBVGh, Blancato and Magni, 2010). This plasmid was constructed using *E. coli* EC101 as host (Blancato and Magni, 2010). Oligonucleotides used for the amplification of *gdpP* gene upstream region were UJ5, AAACCATGGCCGTTTGGGCAATTGAAGACA and UJ6, TTTAAGCTTATTAAAACGGATGACCCCAATTG and for the downstream region were DJ7, AAAAAGCTTATTATGGAGCAAATGGGTGGG and DJ8, TTTCCATGGGCTTTTCTTTTTCCTTAGCTTTGG (**Figure 1C**). Specific gene deletion was confirmed by PCR and the following oligonucleotides: external region of *gdpP:* EJ6, GGTTCTATGAAATTTAAAGCAGTGATTT and EJ7, TTAGGCCTCGCTAATTTTGACTT; internal fragment of *gdpP* IJ4, AAAATGCGAGCGATGACCAA and IJ5, TTAATGGCTGTTCGACCGCT (**Figure 1C**; further details described in Blancato and Magni (2010).

**FIGURE 2:**
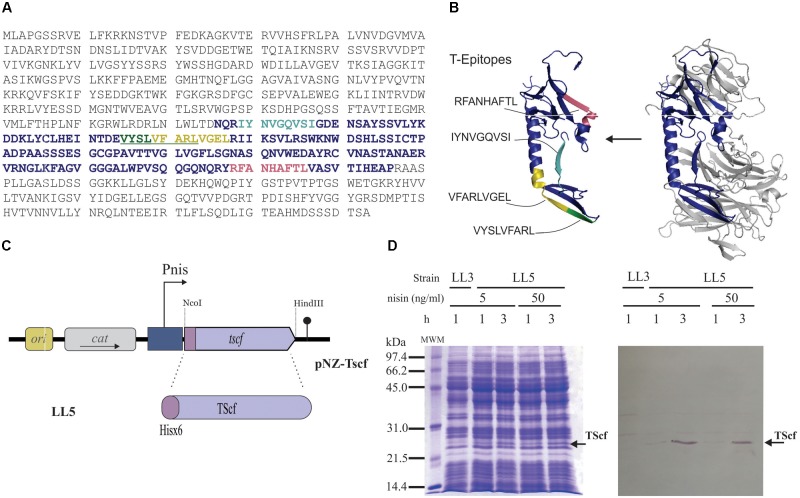
Development of an optimized TS derived antigen. **(A)** Complete sequence of trans-sialidase protein(GenBank: PBJ79959.1). The selected fragment is highlighted in purple and colored amino acids within this sequence indicate predicted T epitopes, underlined amino acids refer to overlapping epitopes. **(B)** Structure modeling of whole TS (right) and the synthetic antigen (left) using PDB entry 1MS3 as template (according to Blancato et al., 2016); Epitopes T are indicated, RFANHAFTL (pale red), IYNVGQVSI (cyan), VYSLVFARL (green) and VFARLVGEL (yellow). **(C)** Cloning representation of his_6x_-*tscf* in pNZ8048 derived plasmid. **(D)** Expression check by SDS-PAGE (left) and Western blot analysis (right). *L. Lactis* NZ9000 clpP-htrA transformed with pNZ8048 (strain LL3) or pNZ-TScf (strain LL5), lactococcal cells were grown in M17G and induced at OD_600_ = 0.5. Nisin concentration and time are indicated in the figure. The band corresponding to Tscf is indicated by an arrow. MWM: low-range molecular weight marker (Bio-Rad, Hercules, CA, United States)

TScf encoding gene cloned in pUC57 was obtained from digestion using *Nco*I and *Hind*III enzymes and subcloned in pNZ8048 plasmid (de Ruyter et al., 1996). Plasmid pNZ-TScf (**Figure 2C**) was electroporated into *L. lactis* strains and positive clones were identified by colony PCR using primers CGAGCATAATAAACGGCTCTG and ATTGCCATTTCAATTGAACG and sequencing (University of Maine, DNA sequencing Facility, United States) (**Table 1**). Plasmid pIQ10-TS carrying both genes encoding *tscf* and *cdaA* in single vector was constructed as follows: *tscf* under control of Pnis promoter region was amplified by PCR using pNZ-TScf as template and the pair of primers IQ696 (AAACTGCAGGTTGAAGAAGGTTTTTATATTACAGC, underlined nucleotides indicate the *Pst*I site) and IQ697 (TTTGTCGACGGTGGACAAATTTACATTAGTCTC, underline indicate the *Sal*I site). The resulting fragment was purified, digested with *Pst*I and *Sal*I, and ligated into the same sites of pIQ101 plasmid (**Figure 4A**). This construction was transformed in *E. coli* DH5αand primary sequence was checked by sequencing (University of Maine, DNA sequencing Facility, United States). Plasmid pIQ10-TS was then electroporated in *L. lactis* cells giving the strain LL7 (**Table 1**).

**FIGURE 3:**
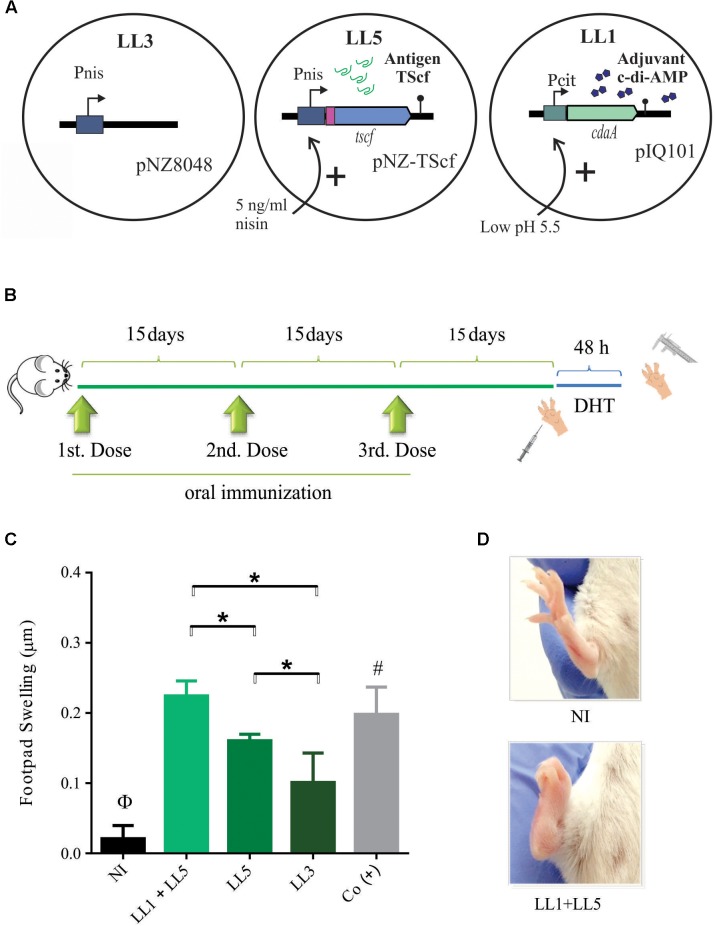
| Oral co-administration of *L. lactis* expressing TScf encoding gene and *L. lactis* overproducing c-di-AMP induce a specific cellular immune response. **(A)** Schematic representation of recombinant *L. lactis* strains used in the experiment. LL1 (induced at pH 5.5 units) and LL3 and LL5 (induced with 5 ng/ml nisin). **(B)** Oral immunization scheme. Three doses were administered with 15 days intervals. Footpad swelling was measured 48 h after the last immunization to determine the degree of delayed-type hypersensitivity (*n* = 4–5 animals/group). **(C)** Immunization carried out by co-administration of LL1+LL5 shows a significant difference with respect to LL5 as well as LL3 groups. **(D)** Morphological difference in footpad swelling in non-immunized mice (up) and LL1+LL5 group (down). NI: non-immunized, Co (+): positive control group. Results are expressed as the difference in footpads thickness after and before the inoculation. ^∗^*p* < 0.05; ^Φ^*p* < 0.05 NI versus the rest of the groups. ^#^*p* < 0.05 among Co (+) and LL5 and LL3.

**FIGURE 4:**
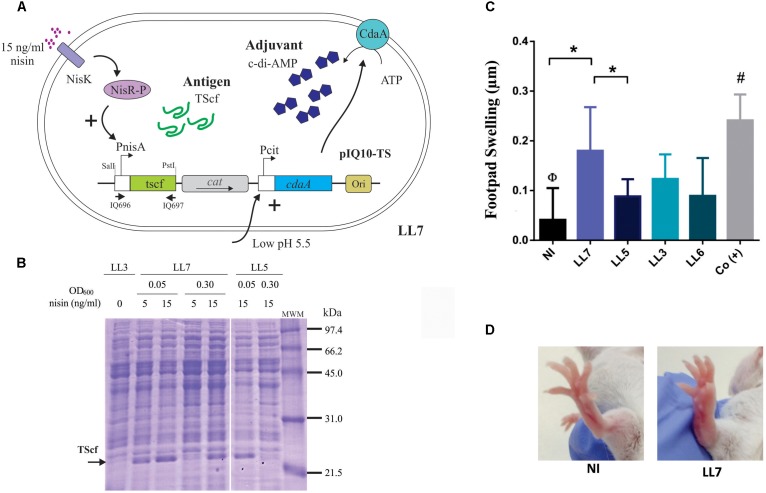
Expression performance and specific immune response induced after oral immunization of *L. lactis* strain simultaneously expressing TScf encoding gene and overproducing c-di-AMP. **(A)** The regulation systems of *L. lactis* NZ9000 *clpP-htrA* transformed with pIQ10-TS (strain LL7). The systems governing the expression of the antigen *tscf* gene (under PnisA control) and *cdaA* gene (under PcitM control) are depicted. **(B)** Production of TScf in crude extracts of engineered *L. lactis* strains. *L. lactis* NZ9000 *clpP-htrA* strain transformed with pNZ8048 (LL3), pNZ-TScf (LL5), or pIQ10-TS (LL7) was grown in M17G at initial pH 5.5 (induction condition for PcitM). Nisin concentrations and OD_600_ used for induction are indicated in the figure. Cells were harvested at OD_600_ = 0.5 in all cases. Arrows indicate TScf band, MWM: low-range molecular weight marker (Bio-Rad, Hercules, CA, United States). **(C)** Footpad testing. Strains LL3, LL5, LL6 (*L. lactis* NZ9000 *clpP-htrA* transformed with pIQ101), and LL7 were grown in M17G at initial pH 5.5 with 15 ng/ml nisin (induction condition for PcitM and PnisA, respectively). The immunization protocol consisted in 3 doses of each strain separated by 15 days intervals. Fifteen days after last immunization, the specific cellular response was analyzed by DHT test (*n* = 4–5 animals/group). Results are expressed as the difference in footpad thickness before and 48 h after TS inoculation. **(D)** Morphological differences in footpads swelling in non-immunized mice (NI) and LL7 group. ^∗^*p* < 0.05; ^Φ^*p* < 0.05 NI versus the rest of the groups. ^#^*p* < 0.05 among positive control group -Co(+)- and LL5, LL3, and LL6.

### Protein Expression and TScf Purification

The His-tagged TScf protein in pNZ-TScf was overexpressed in *L. lactis* NZ9000 *clpP-htrA* strain (Cortes-Perez et al., 2006) (LL5 strain, **Table 1**). Cells were grown in 3 l of M17G broth at 30°C to an OD_600_ = 0.5. Gene expression was induced with 5 ng/ml of nisin and the cells were further incubated for 3 h (**Figure 2D**). Cells were then collected by centrifugation and stored at -80°C. For protein purification, cells were resuspended in lysis buffer (30 mM Tris–HCl pH 8.0, urea 8 M) and were lysed with a mini-beadbeater-16 (Biospec, Bartlesville, OK, United States) using 0.1 μm glass beads. The lysate was clarified by centrifugation, then NaH_2_PO_4_ and imidazole were added to a final concentration of 100 and 5 mM, respectively, pH was adjusted to 8.0. The clarified lysate was run through a Ni^2+^-NTA affinity column (Qiagen) and incubated at room temperature for 1 h to allow binding. Then, the protein was refolded by successive passaged in-column incubation with 50 mM Tris–HCl pH 7.4, 500 mM NaCl, 5% glycerol buffer (buffer C) containing decreasing concentrations of urea ranging from 6 to 0 M. The column was washed with buffer C plus 25 mM imidazole and the protein was eluted from the column in elution buffer (buffer C with 500 mM imidazole). The purified protein was dialyzed against PBS plus 5% glycerol; aliquots were kept at -80°C.

### Protein Extraction and Western Blot Analysis

Protein samples were prepared from 5 ml of *L. lactis* cultures. Cell pellets were washed once with 30 mM Tris–HCl pH 8.0, 150 mM NaCl. Next, bacterial cells were resuspended in lysis buffer (30 mM Tris–HCl pH 8.0, Urea 8 M) and were lysed with a mini-beadbeater-16 (Biospec, Bartlesville, OK, United States) using 0.1 μm glass beads. Protein concentration was determined by Lowry method using bovine serum albumin (BSA) as standard (Lowry et al., 1951).

SDS-PAGE was used to analyze samples, loading 30 μg of total protein per lane in the gels. Protein sizes were estimated using low-range molecular weight marker (Bio-Rad, Hercules, CA, United States). For western blot analysis, proteins were transferred to nitrocellulose membranes using a mini-protean 2 cell unit (Bio-Rad, Hercules, CA, United States). Protein transfer efficiency was assessed by staining with Ponceau red S (Sigma, United States). TScf was detected with anti-his polyclonal antibodies (Santa Cruz Biotechnology, United States) at a 1:200 dilution. Alkaline phosphatase-conjugated goat anti-rabbit immunoglobulin G (Bio-Rad, Hercules, CA, United States) diluted 1:3000 was used as secondary antibody. P-nitroblue tetrazolium chloride (NBT) and 5-bromo-4-chloro-3-indoyl phosphate (BCIP) were used as substrates to detect phosphatase activity.

### Determination of c-di-AMP Intracellular Levels

Twenty milliliter cultures of *L. lactis* were grown in M17G medium supplemented with the corresponding antibiotics when needed and the initial pH indicated in the **Figure 1D**. When samples reached OD_600_ = 0.5, cells were harvested at 4°C and 5000 rpm and quickly frozen in liquid nitrogen. Two additional samples of 1 ml were taken for normalization purposes. Samples were collected and stored at -20°C until c-di-AMP extraction was performed. For this, pellets were resuspended in 150 μl of 2 mg/ml lysozyme in TE buffer and incubated for 30 min at 25°C. Afterward, samples were frozen in liquid nitrogen and boiled at 95°C for 10 min. First, an extraction with 800 μl acetonitrile:methanol 1:1 was performed. Then, two consecutive extractions with 200 μl acetonitrile:methanol:water 2:2:1 were performed. Supernatants were collected and dried in a Speedvac at 40°C. Pellets were sent to Prof. Volkhard Kaever from the Medizinische Hochschule, Hannover for c-di-AMP quantification. Final data was normalized with respect to the amount of protein present in the sample, determined via Lowry assay (Lowry et al., 1951).

### Mice and Animal Facility Conditions

BALB/c female mice, aged 6 weeks, were acquired and housed at the animal facility of the CIPREB (Center for Research and Production of Biological Reagents, School of Medicine, National University of Rosario, Argentina). Mice were housed in HEPA-ventilated racks, 21–22°C and 68% of humidity. Animals had free access to food and water and were maintained under a 12 h light/dark period. All protocols for animal studies were approved by the Bioethics and Animal Care and Use Committees according to Institutional guidelines (Resolution N°6698/2014).

### Preparation of Live Bacterial Inoculum and Immunization Protocol

Three liters of fresh M17G were inoculated with the strain of interest and the corresponding antibiotics at an initial OD_600_ of 0.05. Antigen production (TScf) in strains *L. lactis* LL5 and LL7 was induced at t_0_ by addition of nisin prior to inoculation, concentration is described in each case **Figures 3**, **4**. Synthesis of the adjuvant c-di-AMP (strains LL1, LL6, and LL7 carrying the *cdaA* gene under the promoter region Pcit) was induced by culturing bacteria at initial pH of 5.5 (Marelli and Magni, 2010). Growth was performed at 30°C without shaking until final OD_600_ reached 0.5.

In all cases, cells were harvested by centrifugation at 5000 rpm and 4°C. Pellets were then washed and resuspended in sterile PBS to reach final concentrations in the order of 1 × 10^9^ CFU/100 μl. Afterward, BALB/c female mice were used to evaluate the specific anti-TS cellular immune response of the different engineered *L. lactis* strains. Briefly, mice (*n* = 5 animals/group) were immunized by oral route in three successive doses separated by 2-week intervals. The bacterial dose administered was set as a quantity of bacteria expressing 10 μg of TScf (0.3–1 × 10^6^ CFU/100 μl). Similar quantities of bacteria producing only TScf, CdaA or carrying the vector were administrated by oral gavage using a cannula in parallel groups (100 μl/mice). Taking into account our previous experience on the high efficacy of TS antigen to protect against *T. cruzi* infection when it is delivered subcutaneously, we introduce in parallel a comparative group of animals that were immunized subcutaneously with 10 μg of purified TScf adjuvated with 3 μg of ISPA as a g*old standard* or positive control group [Co(+)], being ISPA an ISCOMATRIX type adjuvant (Bertona et al., 2017).

### Delayed-Type Hypersensibility Response in Mice

To test cellular response, mice were challenged with 5 μg of purified TScf by intradermal injection in the right footpads 12 days after the last immunization. The thickness of hind footpads was measured 48 h after the antigen injection with a digital Vernier caliper. Results of the delayed hypersensitivity test were expressed as the difference in thickness of footpads after and before the inoculation.

### Statistical Analyses

Data analysis were performed using non-parametric tests (Kruskall-Wallis test for the analysis of k < 2 groups while the Mann-Whitney test was employed to analyze differences between two particular groups. All analyses were performed using GraphPad Prisma 6.0 software (GraphPad, La Jolla, CA, United States). The data were considered significant when *p* < 0.05.

## Results

### Construction of a *L. lactis* Strain With High Cytoplasmic Concentration of c-di-AMP

In order to increase the intracellular levels of c-di-AMP in *L. lactis* different strategies were conducted. First, homologous expression of *cdaA*, in charge of c-di-AMP synthesis in *L. lactis* (Reuss et al., 2017) was performed. To do this*, cdaA* was amplified and cloned in the pBV153 vector, resulting in plasmid pIQ101 (**Figure 1A** and **Table 1**). pBV153 was developed in our laboratory and it has the Pcit promoter upstream of the multiple cloning site, leaving the expression of the gene of interest under pH regulation (Marelli and Magni, 2010). pIQ101 plasmid was electroporated in *L. lactis* IL1403, originating *L. lactis cdaA*^+^ (LL1, **Table 1**). Phenotypic impact of the induction of *cdaA* expression was evident on growth curves performed in the rich-medium M17G. *L. lactis cdaA*^+^ needed approximately four additional hours of growth to reach similar μ_max_ and final biomass than the control strain *L. lactis* pBV153 (LL0 strain) (**Figure 1B** and **Table 1**). Changes in growth patterns were more evident in presence of different stress factors. *L. lactis cdaA*^+^ showed a saline hypersensitivity growth defect at 0.25 M NaCl or upon addition of antibiotic compounds (Ampicillin 0.25 μg/ml, Penicillin 0.10 μg/ml, Vancomicyn 0.50 μg/ml), or Lysozyme 0.10 μg/ml (Quintana, 2018). These results suggest that overproduction of CdaA mediates an increment of the intracellular synthesis of c-di-AMP that was previously related to the observed phenotypes in *L. lactis* and other bacteria (Smith et al., 2012; Gundlach et al., 2015; Rismondo et al., 2016; Quintana, 2018).

A second strategy used in order to increase c-di-AMP intracellular concentrations was to inactivate *gdpP.* This gene codes for the unique c-di-AMP phosphodiesterase reported in *L. lactis* to be involved in the degradation of this compound (Smith et al., 2012). The mutant strain where *gdpP* gene was removed via homologous recombination was constructed using the thermosensitive plasmid pIQ095 (**Table 1** and **Figure 1C**). Interestingly, the resulting *L. lactis gdpP^−^* mutant (LL2 strain, **Table 1**) showed normal growth in M17G media. On the other hand, growth parameters were reduced in the presence of the β lactamic antibiotic penicillin, suggesting alteration in the intracellular level of the c-di-AMP of the *L. lactis gdpP*^−^ strain (Quintana, 2018).

With the aim of determining the direct effect of *cdaA* overexpression or *gdpP* disruption on the intracellular levels of c-di-AMP, measures of its concentration were performed in *L. lactis* cultures. Induction at low or neutral initial pH were performed as previously described (Marelli and Magni, 2010). c-di-AMP concentrations in *L. lactis* IL1403 wild type or *L. lactis* pBV153 (LL0) strains were 27 ± 4 and 32 ± 2 ng per mg of protein when initial pH values were set at 7.0 and 5.5, respectively. On the other hand, c-di-AMP concentrations in *L. lactis cdaA*^+^ (LL1) were 342 ± 89 and 675 ± 258 ng per mg of protein at pH 7.0 and 5.5, respectively. As regards *L. lactis gdpP^−^* (LL2), it showed only twice the concentration of c-di-AMP (73 ± 6 ng per mg of protein) at pH 7.0 compared to the wild type strain (**Figure 1D**). These results suggest that the wild type growth phenotype of *L. lactis gdpP^−^* might derive from the mild modification in cytosolic c-di-AMP levels in such mutant. Also, they confirm that *cdaA* gene under Pcit control was induced and generated the accumulation of cytosolic c-di-AMP in *L. lactis cdaA*^+^. Thus, the later strain (LL1), growing at initial pH value of 5.5, where the highest concentrations of c-di-AMP were measured, was selected for its evaluation as immune stimulator.

### Antigen Design and TScf Gene Expression in *L. lactis*

In order to ensure its production in *L. lactis*, the smallest possible protein size of the TS with the highest presence of epitopes able to trigger a TS-specific immune response was selected. Protein regions with the highest density Class I–Restricted T Cell epitopes were selected taking into account that *T cruzi* is an intracellular parasite, and therefore, an immune T cell response is needed to protect against this infection. Since BALB/c mice was our animal model, T epitopes against H-2K^d^ MHC-I were predicted using the tools Propred I (Singh and Raghava, 2001). Four out of the seven epitopes identified by Propred I are localized in the central region of the protein ranging from amino acid 326–496 (**Figure 2A**). Interestingly, the predicted IYNVGQVSI epitope, located in this region, was described as the main MHC-I T-cell epitope that provides protection against *T. cruzi* infections in BALB/c mice (Martin et al., 2006; Rosenberg et al., 2010; Eickhoff et al., 2011). Based on epitope analyzes, the fragment that covers the amino acid 326–496 was selected for immune response studies and called TScf (**Figure 2**).

A TScf encoding gene was synthetized optimizing its codon usage for *L. lactis* and incorporating a stop codon, the *Nco*I and *Hind*III restriction sites required for cloning, and 6xHis encoding codons at 5′ to allow detection by western blot (GenScript, Township, NJ, United States). The synthetic gene was subcloned into pNZ8048, resulting in vector pNZ-TScf (**Figure 2C**) that encodes TScf under the transcriptional control of Pnis promoter. pNZ-TScf was electroporated in *L. lactis* NZ9000 originating strain LL4 (**Table 1**). However, no production of TScf was detected in this host.

An alternative *L. lactis* NZ9000 derived strain used for high level of heterologous proteins production is *L. lactis* NZ9000 *clpP-htrA* strain which is deficient for the two lactococcal major proteases (Cortes-Perez et al., 2006). Then, NZ9000 clpP-htrA strain was transformed with pNZ-TScf, resulting in LL5 strain (**Table 1**). LL5 showed stable overexpression of the TScf encoding gene. As shown in **Figure 2D**, antigen production was barely detectable after 1 h of induction with 5 or 50 ng/ml of nisin but an overproduced band was observed at 3 h with coomasie blue staining. This was confirmed by western blot using anti-his antibodies (**Figure 2D**) whereas protein identity was determined by Mass spectrometry (MS/MS).

### Immune Response Induced by Mucosal Co-administration of *L. lactis* Expressing TScf Encoding Gene and *L. lactis* Overproducing c-di-AMP

Once obtained a strain of *L. lactis* expressing TScf encoding gene and a strain producing high amounts of c-di-AMP, our first aim was to evaluate the potential effectiveness of their co-administration, as proof of concept for the development of a new prototype of mucosal vaccines (**Figure 3A**). Three successive oral immunizations were performed (**Figure 3B**). The studied groups were: (i) NI (non-immunized group -NI-), mice that received only PBS buffer; (ii) LL1+LL5 group, mice co-administered with both induced systems in separated strains, c-di-AMP adjuvant and TScf antigen, respectively; (iii) LL5 group, mice that received *L. lactis* expressing the TScf antigen (**Figure 3**). In addition, *L. lactis clpP-htrA* harboring the pNZ8048 vector (LL3 strain) was also orally administered as control (LL3 group), and finally a group of mice was simultaneously immunized by subcutaneous way with purified TScf adjuvanted with ISPA as a positive control group -Co(+)-, being ISPA a cage like particle adjuvant developed by Dr. Marcipar et al. (Bertona et al., 2017).

As shown in **Figure 3C**, 15 days after the last immunization, all groups [including the Co(+) group] was footpad testing. After 48 h, DHT showed that *L. lactis* LL1+LL5 immunized group elicited a similar magnitude of footpad thickness than Co(+) group. In addition, the TS-specific response elicited by the LL7 group were more evident than in the LL5 group and even greater when compared to NI and LL3 groups. These results support that TScf sequence contains MHC-I T-cell epitopes, but also suggest that orally administered *L*. *lactis* over-expressing *cdaA* gene (LL1) could be used as immune stimulator of the response against *T. cruzi*.

### Engineered *L. lactis* Co-producing Antigen and Adjuvant for Mucosal Administration

In order to construct a fully integrated mucosal vaccine prototype, a single vector carrying both genes encoding the TScf antigen and the CdaA enzyme was designed (**Figure 4**). For this, the TScf encoding region from pNZ-TScf was amplified, including the Pnis promoter and the terminator (**Figure 2A**). The fragment was subcloned in the *Pst*I-*Sal*I restriction sites of vector pIQ101 (**Table 1**). This plasmid was electroporated in *L. lactis clpP-htrA*, and the resulting *cdaA^+^-tscf^+^* strain was named LL7 (**Figure 4A**). Then, *tscf* expression under conditions previously proven to increase c-di-AMP levels in *L. lactis* was evaluated (strain LL5). Hence, *L. lactis cdaA^+^-tscf^+^* (LL7) was grown in M17G medium at initial pH value of 5.5 and *tscf* expression was induced by adding nisin at the initial time, prior to inoculation (OD_600_ = 0.05) or at OD_600_ = 0.3. As shown in the **Figure 4B**, *L. lactis cdaA^+^-tscf^+^* overproduces TScf when 15 ng/ml nisin were added to the media independently of the OD of induction. On the other hand, overproduction of TScf in *L. lactis cdaA^+^* was only detected when nisin was added at OD_600_ = 0.05 (**Figure 4B**).

To analyze the *in vivo* cell-mediated immune response elicited by *L. lactis* co-producing TScf and CdaA (LL7), a similar scheme of three successive oral immunizations was performed, as previously described in **Figure 3B**. *L. lactis* strains expressing the TScf encoding gene (LL5), harboring the vector pNZ8048 (LL3) and *cdaA* (LL6) were also included. Negative and positive control groups were also simultaneously evaluated [NI and Co(+) groups, respectively] (**Figure 4C**). Fifteen days after the last immunization, the degree of inflammation after 48 h of intradermal inoculation of purified TScf was tested (**Figure 4D**). Noteworthy, only LL7 group elicited a TS-specific cellular response of similar magnitude than Co(+). Moreover, was observed a clear increase in the footpad thickness in LL7 group compared to NI or LL5 groups. In addition, the cellular response noticed in LL3 was smaller than that registered in LL7, although it did not reach statistical significance (*p* < 0.06). Moreover, in this case LL5 and LL3 did not differ among themselves. These results indicate that immunization with *L. lactis cdaA^+^-tscf^+^* was effective for sensitizing against TScf.

## Discussion

Vaccination is one of the most important interventions in the field of public health. Molecular techniques opened the possibility to develop vaccines using purified fragments of proteins and recombinant antigens. Nevertheless, these fragments of antigen usually show poorly immunogenic properties and the use of adjuvants becomes necessary to potentiate the specific immune response. Since several pathogens used diverse mucosal surfaces as an entry portal, the development of innovative mucosal vaccines is a priority challenge, even more if the ability of this type of vaccine to elicit both mucosal and systemic immune protection is considered.

On the other hand*, L. lactis* is a good candidate for the delivery of biologically active immunomodulatory proteins or the production of active biological compounds (Wells and Mercenier, 2008). Also, *L. lactis* safety is well established and this microorganism offers a substantial potential as a delivery vector system for vaccines, particularly because it can be administrated by diverse mucosal routes like oral, nasal or intravaginal, and it survives the passage through the gastrointestinal tract as well (Wells and Mercenier, 2008). Here we describe first a live vaccine prototype composed of two strains that showed to elicit a clear TS-specific cell-mediated immune response. One strain (*L. lactis* LL1) of the prototype serves as immune stimulator overproducing the adjuvant c-di-AMP more than 19 times above wild type levels in response to medium acidification (**Figure 1D**). A second antigenic strain (*L. lactis* LL5) overproduces the TScf antigen under control of a nisin inducible expression system. A similar bipartite strategy was used successfully in the development of an intra-nasal vaccine against the human papilloma virus, where one strain expressed the virus antigen and another IL-12 as an immunostimulatory molecule (Bermudez-Humaran et al., 2003). Noteworthy, both approaches evoked an evident cellular response, which likely contribute to the specific Th1-immune response. Moreover, an analogous strategy was also used for desensitization in an experimental allergic airway disease model (Cortes-Perez et al., 2006, 2007). Despite DHT as an estimation of TS-specific cellular response has same limitations (i.e., does not allow to recognize the T subpopulations involved in the specific response or the cytokines contributing in such reaction), the DHT assay continue to be one of the most rapid and available tests for the evaluation of this type of response during the screening of vaccine prototypes. As we have previously shown in other immunization schemes using TS (Bontempi et al., 2015, 2017; Bertona et al., 2017), it is expected that IFN-γ be one of the cytokines involved in this type of reaction.

In this work, we also showed for the first time that a single *L. lactis* strain producing both the c-di-AMP adjuvant and a heterologous antigen (TScf), was capable to elicit a better specific immune response compared to a *L. lactis* strain producing only the antigen. The co-existence of both molecules in the same strain of *L. lactis*, not only may favor the development of a specific immune response (by exposing immunocompetent cells to both molecules at the same time), but it can help as well to reduce costs for the implementation of vaccination programs in developing countries. In fact, other cases were reported, where one-strain strategies were used, involving *L. lactis* strains expressing a fusion protein of two antigens or an antigen and the peptidic IL-2 adjuvant (Zhang et al., 2014; Beck et al., 2017). Interestingly, one-strain vaccine prototypes based on a *L. lactis* that overproduced adjuvants could be used or combined with other antigens enabling systematic research of a variety of antigens.

c-di-AMP exerts its adjuvant effect triggering a balanced Th1/Th2/Th17 response and a strong IFN-type I production via the STING-TBK1-IRF3 cascade (Burdette et al., 2011; Burdette and Vance, 2013). Very promising results were obtained when c-di-AMP was assessed as adjuvant in different prototypes of mucosal vaccines against different viruses and bacteria (Sanchez et al., 2014; Landi et al., 2017; Schulze et al., 2017). Particularly, this adjuvant has also been used in previous studies for the design and experimental assessment of subunit vaccines formulations against *T. cruzi* (Matos et al., 2017; Sanchez Alberti et al., 2017). In these studies, recombinant *T. cruzi* antigens were formulated together with c-di-AMP and were administered nasally, obtaining an immune response that allowed protection after the challenge with the parasite. Moreover, using the Tc52 *T. cruzi* antigen, Matos and colleagues described a better adjuvant ability of c-di-AMP in comparison with CpG, one of the most potent adjuvants for the development of vaccines against intracellular microorganisms (Matos et al., 2017). Reinforcing these data, our results also show that an engineered *L. lactis* that overexpresses c-di-AMP and a TS fragment could result in an effective vaccine for Chagas disease.

Nowadays, c-di-AMP is only produced by expensive and laborious procedures (Zheng et al., 2013). Engineering of *L. lactis* overproducing c-di-AMP can solve this problem, allowing to reach adequate quantities at mucosal level. However, the design of a *L. lactis* strain with high intracellular concentration of c-di-AMP is a rewarding but also a daunting task due the fact that unbalanced intracellular levels of c-di-AMP might prevent or hinder *L. lactis* growth. In fact, during the design and evaluation of the c-di-AMP overproducer *L. lactis* strain, several combinations of promoters with different strengths (Pnis, Pcit), *cdaA* homologs (from *E. faecalis* or *L. lactis)*, and hosts with different genetic backgrounds (wild type, *htrA^−^ clpP^−^*, or *gdpP^−^*) were evaluated (not shown). Remarkably, in the present study the objective to obtain a c-di-AMP overproducer *L. lactis* strain with immune stimulatory properties was fulfilled. Nevertheless, further studies should be performed to broaden the knowledge regarding the regulation of c-di-AMP synthesis and degradation, as well as its role in the physiology of *L. lactis.* This will open new opportunities in the development of oral and mucosal vaccines.

## Author Contributions

CM, VB, ME, AP, and IM contributed conception and design of the study. VB, ME, IQ, and CM made genetic experiment. CM, VB, ME, IM AP, and JS organized the database. FG, SV, MP, and AP made *in vivo* experiment. SV, FG, FP, GC, EP, AP, and IM performed the statistical analysis and immune response sections of the manuscript. CM, AP, and IM wrote the first draft of the manuscript. IQ, ME, VB, and CM wrote engineering lactococcal sections. All authors contributed to manuscript revision, read and approved the submitted version.

## Conflict of Interest Statement

The authors declare that the research was conducted in the absence of any commercial or financial relationships that could be construed as a potential conflict of interest.
